# THE EFFECT OF A DRUG-BURDEN INDEX ON FALLS RISK INCREASING MEDICATIONS DEPRESCRIBING IN VETERANS AT RISK OF FALLING

**DOI:** 10.1093/geroni/igad104.3633

**Published:** 2023-12-21

**Authors:** Michelle Paulsen, Sunny Chen, Erica Martinez, Katherine Ritchey

**Affiliations:** VA Puget Sound, Seattle, Washington, United States; VA Puget Sound, Seattle, Washington, United States; VA Puget Sound, Seattle, Washington, United States; University of Washington, Seattle, Washington, United States

## Abstract

Falls risk-increasing medications (FRIMs) have negative effects on balance and psychomotor functioning which increase the risk of falling in older adults. Thus, deprescribing of FRIMs is a recommended fall prevention intervention. The Drug Burden Index (DBI) measures total exposure to anticholinergic and sedative medications and has been associated with falling risk in older adults. We evaluated if inclusion of a DBI score would influence deprescribing of FRIMs in older veterans at risk of falling. A pharmacist conducted a medication review for FRIMs in two cohorts of older veterans participating in a fall prevention group. The primary care prescriber (PCP) was alerted to recommendations through the electronic medical record. Inclusion of the DBI was included and shared with the PCP in the second cohort only. Changes to FRIMs and reduction in DBI score was calculated for all participants three months after initial FRIM review. Nearly all (95%) participants (n=44) were prescribed a FRIM. The most common active prescriptions contributing to DBI score were for anticholinergics (37.9%), antidepressants (33.3%) or anticonvulsants (31.8%) agents. DBI improved in both cohorts, but DBI change was greatest in the second cohort (n=18) (first cohort mean DBI change= 0.168; second cohort mean DBI change= 0.189; p 0.002). Percentage of recommendations accepted and acted upon by PCP was similar between both groups (60% vs 59%). Inclusion of a DBI is a helpful measure of FRIM deprescribing and may further encourage deprescribing behaviors. Feedback from PCPs will clarify the role of DBI in fall prevention medication reviews.

